# Metabolic impact of weight variations in Icelandic horses

**DOI:** 10.7717/peerj.10764

**Published:** 2021-01-28

**Authors:** Julien Delarocque, Florian Frers, Korinna Huber, Klaus Jung, Karsten Feige, Tobias Warnken

**Affiliations:** 1 Clinic for Horses, University of Veterinary Medicine Hannover, Foundation, Hannover, Germany; 2 Institute of Animal Science, Faculty of Agricultural Sciences, Universität Hohenheim, Stuttgart, Germany; 3 Institute for Animal Breeding and Genetics, Tierärztliche Hochschule Hannover, Hannover, Germany

**Keywords:** Equine metabolic syndrome, Insulin dysregulation, Oral glucose test, Obesity, Metabolomics, Pathway analysis

## Abstract

**Background:**

Insulin dysregulation (ID) is an equine endocrine disorder, which is often accompanied by obesity and various metabolic perturbations. The relationship between weight variations and fluctuations of the insulin response to oral glucose tests (OGT) as well as the metabolic impact of ID have been described previously. The present study seeks to characterize the concomitant metabolic impact of variations in the insulin response and bodyweight during repeated OGTs using a metabolomics approach.

**Methods:**

Nineteen Icelandic horses were subjected to five OGTs over one year and their bodyweight, insulin and metabolic response were monitored. Analysis of metabolite concentrations depending on time (during the OGT), relative bodyweight (rWeight; defined as the bodyweight at one OGT divided by the mean bodyweight across all OGTs) and relative insulin response (rAUC_ins_; defined accordingly from the area under the insulin curve during OGT) was performed using linear models. Additionally, the pathways significantly associated with time, rWeight and rAUC_ins_ were identified by rotation set testing.

**Results:**

The results suggested that weight gain and worsening of ID activate distinct metabolic pathways. The metabolic profile associated with weight gain indicated an increased activation of arginase, while the pathways associated with time and rAUC_ins_ were consistent with the expected effect of glucose and insulin, respectively. Overall, more metabolites were significantly associated with rWeight than with rAUC_ins_.

## Introduction

Insulin dysregulation (ID) is an equine endocrine disorder encompassing insulin resistance (IR) and basal or post-prandial hyperinsulinemia (HI), which predisposes horses for a crippling hoof condition called laminitis (Frank & Tadros, 2014). The oral glucose test (OGT) can be used to diagnose and quantify ID as it seizes both its enteric and systemic component (De Laat, McGree & Sillence, 2015; Bertin & Laat, 2017).

The impact of weight gain or weight loss on IR and ID has been described numerous times (Van Weyenberg et al., 2008; Carter et al., 2009; Morgan, Keen & McGowan, 2016; Bamford et al., 2019), substantiating obesity as a major risk factor for ID (Geor & Harris, 2009; Morgan, McGowan & Mcgowan, 2014) and establishing dietary energy restrictions and exercise programs as main requirements for the management of patients with this condition (Durham et al., 2019).

Because of its central role in energy metabolism, insulin is tied to many molecule classes. For example, amino acids have long been known to exert a regulatory function on β-cells and increase insulin secretion whereas insulin regulates protein synthesis (Floyd et al., 1966; Felig, 1975; Kimball, Vary & Jefferson, 1994). Some amino acids and derived biogenic amines or even broader classes of lipids (e.g., phosphatidylcholines, lysophosphatidylcholines and sphingomyelins) have been associated with specific pathomechanisms of metabolic conditions (Newsholme et al., 2007; Holland et al., 2008; McKnight et al., 2010). Therefore, metabolomics approaches covering this broad range of molecules have been used for the identification of candidate biomarkers and to investigate the pathophysiology of such conditions or their risk factors (Pallares-Méndez et al., 2016; Lent-Schochet et al., 2019). In contrast to hypothesis-driven approaches, such high-throughput methods aim to describe the studied systems in a global way, including their often complex interactions and capable of discovering unmapped pathways (Kell & Oliver, 2004).

Similar methods have been used in horses with ID, suggesting, for example, an impact of ID on the tricarboxylic acid cycle (Jacob et al., 2018). Previous experiments using the same assay were successful in identifying potential biomarkers of ID but did not include predictors related to bodyweight (Kenéz Warnken, Feige & Huber, 2018; Delarocque et al., 2020b). Besides the effect of weight gain on the lipidome (Blaue et al., 2019; Coleman et al., 2019), little is known about the relationship between obesity and the metabolome in horses. Since the relationship between variations in body weight and IR or ID is well known, an impact of such variations on the metabolites affected by ID is likely. The description of the respectively affected pathways could lead to new hypotheses for the treatment of these conditions. As a result, the objective of this retrospective study was to investigate the interplay between weight variations and changes in the insulin and metabolic response to repeated OGTs in an inductive framework. The main hypothesis was that weight gain and worsening of ID have a distinct metabolic impact during OGT.

## Materials & Methods

The data presented here were obtained from blood samples collected as part of a study describing the relationship between weight variations and insulin response to an OGT (Delarocque et al., 2020a). The study was approved by the State Office for Consumer Protection and Food Safety (LAVES) in accordance with the German Animal Welfare Law (File #33.8–42502–04-17/2646).

### Horses

Nineteen university-owned Icelandic horses of mixed metabolic status from two herds were enrolled in this project. One group included five horses (1 stallion, 4 geldings; median age: 17 years, range: 9–17 years), while the other comprised fourteen horses (11 mares, 3 geldings; median age: 21 years, range: 14–29 years). Both groups had access to neighboring pastures and were fed hay from the same batches.

### Oral glucose tests

Five OGT were conducted at even intervals over one year. The horses were weighed using a mobile weighing scale (accuracy: 1%, resolution: 1 kg, precision: 2 kg) 9 to 16 days prior to each test. The horses were fasted for 12 h. In the morning (8:00–9:00 a.m.), a jugular vein catheter was aseptically placed for blood collection. After a basal blood sample had been drawn, 0.5 g/kg bwt glucose was administered *via* a nasogastric tube. Further blood samples were collected 30, 60, 120, 180 and 240 min later. After collection, the samples were separated into potassium EDTA and Z serum clot activator vacuum tubes (Greiner Bio-One International GmbH, Frickenhausen, Germany). The EDTA tubes were chilled at 4 °C, while the serum tubes were left to clot at room temperature. They were centrifuged at 4,000 g for 10 min within 6 h, for the plasma and serum supernatants to be collected, aliquoted and stored at –80 °C until further analysis.

### Insulin measurements

Serum insulin concentrations were measured in duplicate at the end of the experimental phase using an equine-optimized ELISA (Mercodia Equine Insulin ELISA; Mercodia AB, Sylveniusgatan 8A, Uppsala, Sweden; inter-assay coefficient of variation: 7.7%) previously validated for use in horses.

### Metabolomic assay

Metabolomic profiling of the 0 and 120 min plasma samples was performed at the Fraunhofer Institute of Toxicology and Experimental Medicine ITEM, Hanover, Germany, using the AbsoluteIDQ p180 Kit (Biocrates life sciences AG, Innsbruck, Austria). This assay includes up to 188 metabolites related to glycolysis, oxidative processes, lipid degradation and inflammatory signaling. Amino acids and biogenic amines were measured by liquid chromatography-tandem mass spectrometry while acylcarnitines, hexoses, phosphatidylcholines (PCs), lysophosphatidylcholines (LysoPCs) and sphingomyelins (SMs) were quantified using flow injection analysis-tandem mass spectrometry.

### Statistical analysis

Statistical analysis was performed with R 4.0.0 (R Core Team, 2020). Metabolites with over 50% of values below the limit of detection were discarded. Remaining values below limit of detection were set to limit of detection/2. Missing values were imputed by the *k*-nearest neighbors method (Hastie et al., 1999). Measurement batches were aligned using the QC-RLSC method (Dunn et al., 2011). Metabolites with a coefficient of variation over 20% within the quality control samples were removed from further analysis. Substance class summaries of metabolite concentrations and the kynurenine to tryptophan ratio were computed. Values were then log_2_-transformed, adjusted for measurement and experimental (OGT replicates) batches using the ‘removeBatchEffect’ function from the ‘limma’ package (Ritchie et al., 2015), auto-scaled and quantile-normalized (Bolstad et al., 2003).

The relative weight (rWeight; weight at one OGT divided by the mean weight across all OGTs) and relative area under the insulin curve over time (rAUC_ins_; defined similarly) were used as predictors of metabolite concentrations alongside the time of the OGT in a mixed linear model adjusted for group, age and sex using the ‘limma’ package to investigate the metabolic impact of weight variations and the insulin response.

Metabolite set enrichment analysis (MSEA) was performed using the ‘mroast’ function from the ‘limma’ package. According to the definition of Goeman & Bühlmann (2007), this function provides a self-contained set test relying on the principle of rotation applicable to linear models (Wu et al., 2010). Metabolite identifiers were obtained from the human metabolome database (Wishart et al., 2018) and associated with metabolic pathway identifiers from the small molecule pathway database (Jewison et al., 2014). Long-chain phospholipid concentrations in the p180 assay can represent the sum of several physiologically close isomers. In such cases, the first best match from the human metabolome database was kept as a metabolite identifier. Only pathways including at least three distinct metabolites from the cleaned dataset were kept for analysis.

*P*-values were adjusted for multiple comparisons using the method of Benjamini–Hochberg (Benjamini & Hochberg, 1995). Statistical significance was set at 0.05 (after adjustment for multiplicity).

## Results

### Clinical parameters

The evolution of the insulin response to the OGT and bodyweight during the study period was described previously (Delarocque et al., 2020a). Briefly, the variations in bodyweight were similar in both groups with an overall median maximal weight difference of 43 kg (11%) while the variations in the insulin response differed. On the small pasture a median maximal variation of the AUC_ins_ of 68% was observed, while horses on the large pasture had a median maximal variation of 123%.

Despite a general trend of weight loss over the study period, the horses gained weight between two successive OGTs in 29% of the cases. The insulin response and bodyweight of the horses at each OGT are provided as an additional file (Table S1).

None of the horses developed laminitis or showed any other clinical abnormalities throughout the study.

### Data preparation

Substance class summaries and the kynurenine/tryptophan ratio were added to the 188 metabolites measured by the Biocrates AbsoluteIDQ p180 Kit, resulting in 194 features. After preprocessing, 116 features were still present, as summarized in Table 1.

Nineteen horses were each subjected to five OGTs, for each of which two timepoints were considered in the metabolome, resulting in 190 samples.

### Linear models

The impact of the time during the OGT, rWeight and rAUC_ins_ on the metabolite concentrations was investigated using linear models. The first factor describes the time course of metabolite concentrations during the OGT, the second one represents the impact of variations in bodyweight and the third one shows the influence of changes in the insulin response.

**Figure 1:**
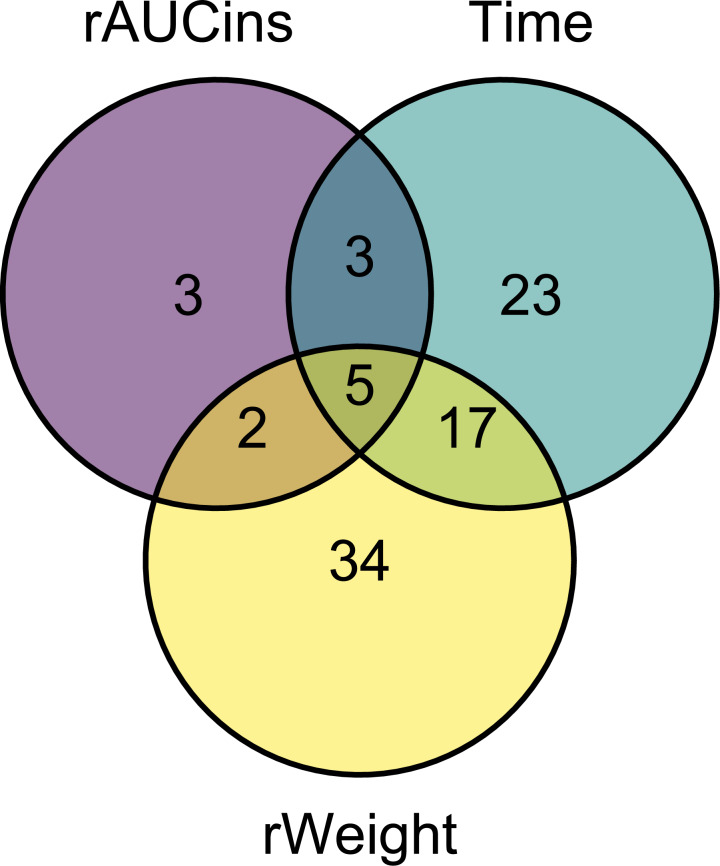
Venn diagram of the metabolites significantly associated with the factors of interest in the linear model. Each circle represents one factor of interest. The number within each region stands for the numbers of metabolites significantly associated with one or more of these factors according to the circles overlapping. As an example, two metabolites were significantly associated with both rAUC_ins_ and rWeight, although the direction of association can vary (positive or negative).

**Figure 2:**
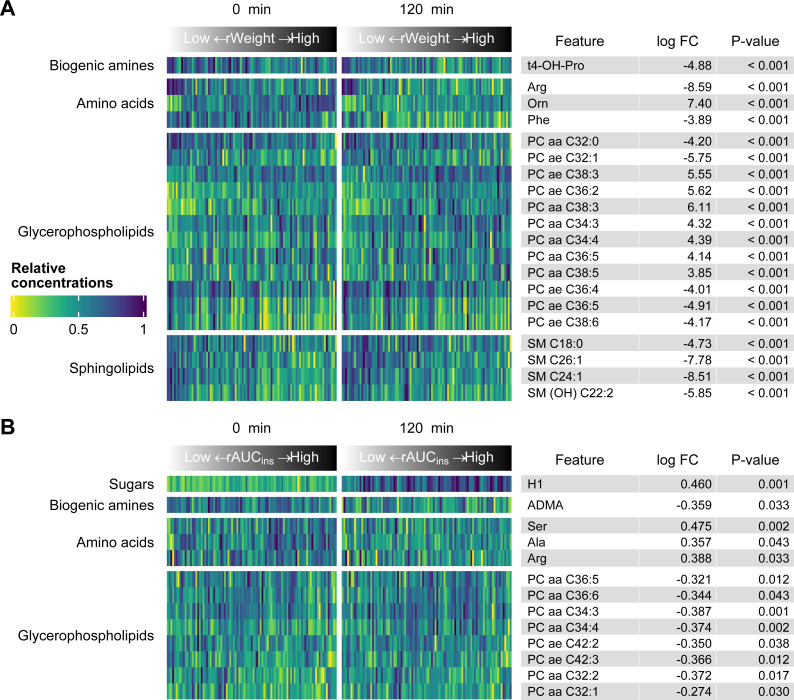
Heatmaps of the metabolite concentrations significantly associated with rWeight (A) and rAUC_ins_ (B). Only the top 20 metabolites are shown for rWeight. The samples are arranged by increasing rWeight or rAUC_ins_ and grouped by the time point of the OGT. This allows one to observe if the impact of the variables of interest is the same at both time points and prevents the effect of rWeight or rAUC_ins_ to be masked by the effect of time (e.g., as would be the case for sugars [H1]).

The number of metabolites significantly associated with each of the factors of interest from the linear model are displayed in Fig. 1. The greatest number of metabolites was associated with rWeight, followed by the effect of time during the OGT. Many metabolites were affected by more than one of these factors but not necessarily in the same direction (i.e., a metabolite might have been negatively associated with rWeight and positively associated with rAUC_ins_, as shown in Fig. 2). The five metabolites affected by all three factors were arginine (Arg), serine (Ser) and the PCs: PC aa C32:1, PC aa C34:3 and PC aa C34:4. Most of the metabolites affected by both rWeight and time were PCs as well. The sum of hexoses is essentially representative of glucose during the OGT and was positively associated withrAUC_ins_ and time.

**Table 1:** Metabolites available before and after data pre-processing. Summarized values are the sums of plasma concentrations of metabolites by groups (e.g., sum of acylcarnitines) or ratios like the kynurenine:tryptohphan-ratio, which is of interest in the scope of inflammatory processes.

Class	Before pre-processing	After pre-processing
Acylcarnitines	40	1
Amino acids	21	20
Biogenic amines	21	11
Glycerophospholipids	90	65
Sphingolipids	15	13
Sugars	1	1
Summarized values	6	5
Total	194	116

Figure 2 visualizes the metabolic impact of rWeight (A) and rAUC_ins_ (B) at each time point using heatmaps. While all metabolites significantly associated with rAUC_ins_ are shown (Fig. 2B), only the top 20 metabolites significantly associated with rWeight are presented (Fig. 2A). Overall, the same patterns are visible at 0 and 120 min, however, the gradient was more pronounced at one of the timepoints for some metabolites (e.g., ornithine (Orn) concentrations increased with rWeight at both timepoints, however, this was more pronounced before oral glucose loading [0 min]). The metabolites predominantly affected were glycerophospholipids. The effect of rAUC_ins_ on this class was exclusively negative and partly opposite to the effect rWeight (e.g., PC aa C36:5). It should be noted that the fold changes, indicating the changes in normalized metabolite concentrations for each unit of rWeight of rAUC_ins_, cannot be directly compared since they are on different scales.

Asymmetric dimethylarginine (ADMA) was negatively associated with rAUC_ins_. By contrast, the amino acids arginine, serine and alanine (Ala) were positively correlated with this factor. Interestingly, arginine displayed a negative association with rWeight, alongside phenylalanine (Phe), trans-4-hydroxyproline (t4-OH-Pro) and four SMs.

### Metabolite set enrichment analysis

**Figure 3:**
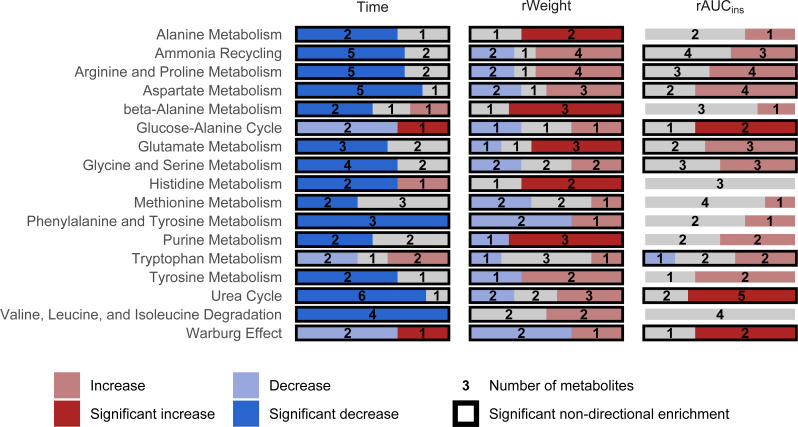
Impact of the three factors of interest from the linear model on the 17 pathways included in MSEA. Time is mostly associated with a decrease in metabolic activity, while the other two factors counterbalance this effect.

Seventeen pathways contained three or more metabolites and were available for MSEA. As presented in Fig. 3, all pathways were significantly, mostly negatively, associated with the effect of time in the OGT. While all pathways were significantly associated with rWeight as well, this effect is more ambiguous, with fewer pathways displaying an obvious positive or negative association. Nevertheless, the metabolism of alanine, glutamate, histidine and purine appeared to be positively associated with rWeight (i.e., more active upon weight gain). As for rAUC_ins_, it had a positive impact on metabolites from the glucose-alanine and urea cycle and a pattern compatible with the Warburg effect. Overall, the effect of rWeight and rAUC_ins_ were opposed to the effect of time.

## Discussion

The metabolic response of 19 horses to five OGTs was investigated while considering the impact of changes in bodyweight and the insulin response. The underlying aim was to illustrate and distinguish the impact of weight gain and an aggravation of ID on the metabolism. Univariate analysis highlighted the impact of these effects on glycerophospholipids. The effects of the relative weight and insulin response in MSEA were opposed to the effect of time in the OGT, which describes the immediate metabolic response induced by the glucose bolus.

### Metabolic impact of variations in bodyweight

Variations in bodyweight were represented by the rWeight, which allowed one to compare the evolution of bodyweight between horses. Positive associations between metabolite concentrations and the rWeight can be interpreted as the metabolic impact of weight gain irrespective of its cause (the same being true for negative associations and weight loss). The quality of feed inducing weight gain affects the extent of ID (Bamford et al., 2016). Moreover, weight loss achieved by dietary restrictions and exercise can provide additional metabolic benefits compared to dietary restrictions alone (Carter et al., 2010; Moore, Siciliano & Pratt-Phillips, 2019; Bamford et al., 2019). Because physical activity energy expenditure and energy intake were not measured, the effect attributed to weight gain or loss in this retrospective study can result from any or both components. Additionally, the metabolic response to the OGT may vary depending on the proportions of metabolically active tissues (e.g., muscle mass *versus* adipose tissue) and their functional integrity (e.g., adipose tissue dysfunction). Neither parameter was assessed in the present study, but both might be affected by variations in bodyweight (Blaue et al., 2019; Reynolds et al., 2019).

The metabolites PC aa C32:0, PC ae C36:2, PC ae C36:4, PC ae C36:5 and PC ae C38:6 have been previously reported to be negatively associated with the body mass index in humans (Wallace et al., 2014), while the metabolites SM C18:0, SM (OH) C22:2, t4-OH-Pro and SM C26:1 were decreased in type 2 diabetes mellitus (Allalou et al., 2016; Isherwood et al., 2017), showing good agreement with the results from the present study. It should be noted that the studies on diabetes mellitus included individuals with a mean body mass index over 30 or a higher mean body mass index in the diabetes group, which was compared to an obese/overweight group, so that a contribution of obesity to the effects observed is possible. Nevertheless, opposite patterns were also described for PC ae C36:2 (Wallace et al., 2014) and SM C18:0 (Hanamatsu et al., 2014). Overall, the similarity of the metabolites associated with obesity in humans and horses suggests the presence of common pathophysiological processes across species.

Decreased t4-OH-Pro has previously been associated with ID in horses (Kenéz Warnken, Feige & Huber, 2018), however, the impact of obesity was not analyzed. Since obesity is a major risk factor for ID, the present results appear to be compatible with the previous findings. Since proline hydroxylation requires the antioxidant ascorbic acid, it was hypothesized that hydroxyproline is an indirect marker of oxidative stress in several species (Kenéz Warnken, Feige & Huber, 2018; Lent-Schochet et al., 2019; Zhang et al., 2020). In addition to indicating oxidative stress, a decrease of t4-OH-Pro upon weight gain might arise from a lack of ascorbic acid secondarily to oxidative processes and result in the production of structurally unstable collagen, which could weaken the lamellar basement membrane. On the other hand, there is contradictory evidence regarding the association of HI or obesity and oxidative stress (Treiber et al., 2009; Pleasant et al., 2013; Banse et al., 2015).

The alanine metabolism was positively associated with weight gain, however alanine itself was not, so that the remaining metabolites are more representative of purine or, more probably, glutamate metabolism. Both glutamate and glutamate metabolism were significantly associated with rWeight. Monosodium l-glutamate was shown to suppress weight gain in rats, possibly by increasing the energy expenditure (Kondoh & Torii, 2008). Moreover, it was reported to increase satiety and reduce voluntary energy intake in humans (Kondoh & Torii, 2008). Therefore, the present findings could indicate a regulatory effect of glutamate metabolism upon weight gain.

Similarly, the impact of rWeight on beta-alanine and histidine metabolism, mainly mediated by glutamate, histidine and carnosine, might result from an adaptation to an increased lipogenesis (Mong, Chao & Yin, 2011).

Variations in arginine concentrations are attributable to its metabolization (among others into nitric oxide (NO), creatinine, ornithine and citrulline), the level of protein synthesis and turnover, de novo synthesis and dietary uptake (Morris, 2016). Nevertheless, based on the present results, it cannot be determined which mechanisms are associated with rWeight and rAUC_ins_, respectively, or if these mechanisms are a cause or a consequence of ID or weight gain. Arginine has a potent vasodilatory effect mediated by NO (Bode-Böger, 2006). Therefore, it is interesting that vascular dysfunction has previously been associated with endocrinopathic laminitis (Morgan et al., 2016), which is the main clinical consequence of ID.

While arginine was strongly negatively associated with rWeight, the opposite was true for ornithine. This implies an inverse association between rWeight and the Arg:Orn ratio, which was reported to be negatively associated with arginase activity (Kashyap et al., 2008; Kövamees, Shemyakin & Pernow, 2016) since Arg is the immediate precursor of Orn in the arginase pathway (Morris, 2016). An increased arginase activity, which is supported by the present results, would result in competitive inhibition of NO synthetase, which could, in turn, affect endothelial function (Sourij et al., 2011).

### Metabolic impact of changes in the insulin response

Changes in the insulin response were assessed using the rAUC_ins_, which is the total insulin response approximated by the area under the insulin curve during the OGT (AUC_ins_), relative to the horse’s mean total insulin response. This measure makes the evolution of the insulin response comparable across horses. An increase in rAUC_ins_ indicates a worsening of ID.

Arginine was positively associated with an increased insulin response (log_2_ fold-change = 0.39); however, in absolute numbers, this relationship was much weaker than the negative relationship between rWeight and arginine (log_2_ fold-change = −8.59). While the scales of rWeight and rAUC_ins_ differ, the difference between the absolute fold changes remains obvious even when adjusted for the relationship reported previously between rWeight and rAUC_ins_, where the impact of rWeight on rAUC_ins_ was fivefold (Delarocque et al., 2020a). As weight gain is often associated with an aggravation of ID (Carter et al., 2010), the impact of rWeight on arginine might prevail on the effect of rAUC_ins_ when only one of these measures is accounted for in the statistical model. Conversely, this result highlights that different metabolic mechanisms appear to be triggered by weight gain and worsening of ID.

Arginine is also known as an insulin secretagogue (Floyd et al., 1966), which might explain why arginine was positively associated with rAUC_ins_ and negatively associated with rWeight.

The rAUC_ins_ was also negatively associated with ADMA. However, the relevance for the pathomechanism of ID or laminitis remains unknown as this molecule inhibits nitric oxide synthesis (Bode-Böger, 2006).

The opposite impact of time and rAUC_ins_ on the urea cycle is consistent with previous reports (Hamberg & Vilstrup, 1994). As expected, the induced hyperglycemia is associated with a decrease in products of the urea cycle, while HI is not. The positive effect of rAUC_ins_ on urea cycle metabolites might be mediated by a reduction of hyperglycemia, which would imply an adequate insulin sensitivity of the liver even in insulin dysregulated horses.

### Considerations on data analysis

Although rWeight has previously been reported to be linearly associated with rAUC_ins_, it should be acknowledged that the correlation between the two was essentially conditional on the Group (see Methods/Animals) (Delarocque et al., 2020a). While the model was adjusted for the effect of Group, the predictors were not. The raw correlation between rWeight and rAUC_ins_ was moderate (*r* = 0.44), but the coefficients associated with the predictors determined for each metabolite were barely affected by the exclusion of the other variable of interest from the model (Fig. S1). As a result, the model used in the present study does not appear to have been affected by collinearity.

It is necessary to map the metabolites to known pathways in order to perform MSEA. This presupposes sufficient knowledge of both the pathways and the metabolites, but this presumption is not fulfilled equally for all metabolites (in contrast to most genes). As an example, glycerophospholipids were largely impacted by both rWeight and rAUC_ins_ but unrepresented in MSEA, which could represent a form of bias.

## Conclusions

The results supported a pro-inflammatory impact of weight gain and suggested that it affects glutamate metabolism. The arginine concentrations were affected in opposite ways by rWeight and rAUC_ins_, potentially inducing vascular dysfunction but also involved in the modulation of the insulin response. Both glutamate and arginine can easily be supplemented orally, warranting the exploration of new adjunct dietary approaches to hamper ID in future studies.

##  Supplemental Information

10.7717/peerj.10764/supp-1 Supplemental Information 1 Raw and preprocessed metabolite concentrations Includes the sample data, feature data and raw and preprocessed concentration data. Rows of the sample data correspond to columns in the concentration data sheets (rownames correspond to column names) and rows of the feature data sheet correspond to the rows of the concentration data sheets.

10.7717/peerj.10764/supp-2 Supplemental Information 2 Insulin response and bodyweight of the horses at each OGT This data was presented in a previous publication describing the relationship between the relative bodyweight (rWeight) and relative insulin response (rAUC_ins_). The present manuscript seeks to correlate this data to the metabolic response to OGT and thereby to describe the interplay between these three variables.

10.7717/peerj.10764/supp-3 Supplemental Information 3 Scatterplot comparing the coefficients associated with rAUC_ins_ and rWeight in the adjusted and unadjusted linear models The adjusted models include both predictors (rAUC_ins_ and rWeight), while the unadjusted models include only one them. The almost perfect agreement (high correlation along the identity line, r ≥ 0.95) shows that the models are not affected by collinearity issues between rAUC_ins_ and rWeight.

10.7717/peerj.10764/supp-4 Supplemental Information 4 Comparison of the raw metabolite concentrations with the human QC samples Boxplots of the raw sample data compared to the medium human QC (lyophilized human plasma with medium concentration levels). The plot is split in three parts (one for each measurement batch/assay plate). The boxplots contain all sample measurements for each metabolite and the human samples (which are technical replicates) are shown on top as red dots. Overall, the patterns are very close among batches and human levels are close to the equine ones a well.

## Additional Information and Declarations

### Data Availability

The following information was supplied regarding data availability: Raw data is available in the Supplemental Files.
