# Supplementary figures and images for: Metabolic impact of weight variations in Icelandic horses
Source: PeerJ. 2021 Jan 28;9:e10764. doi: 10.7717/peerj.10764 (PMC7847705; doi:10.7717/peerj.10764)

$P < 0.05$

Neither

Adjusted model

Both

Unadjusted model

Identity line

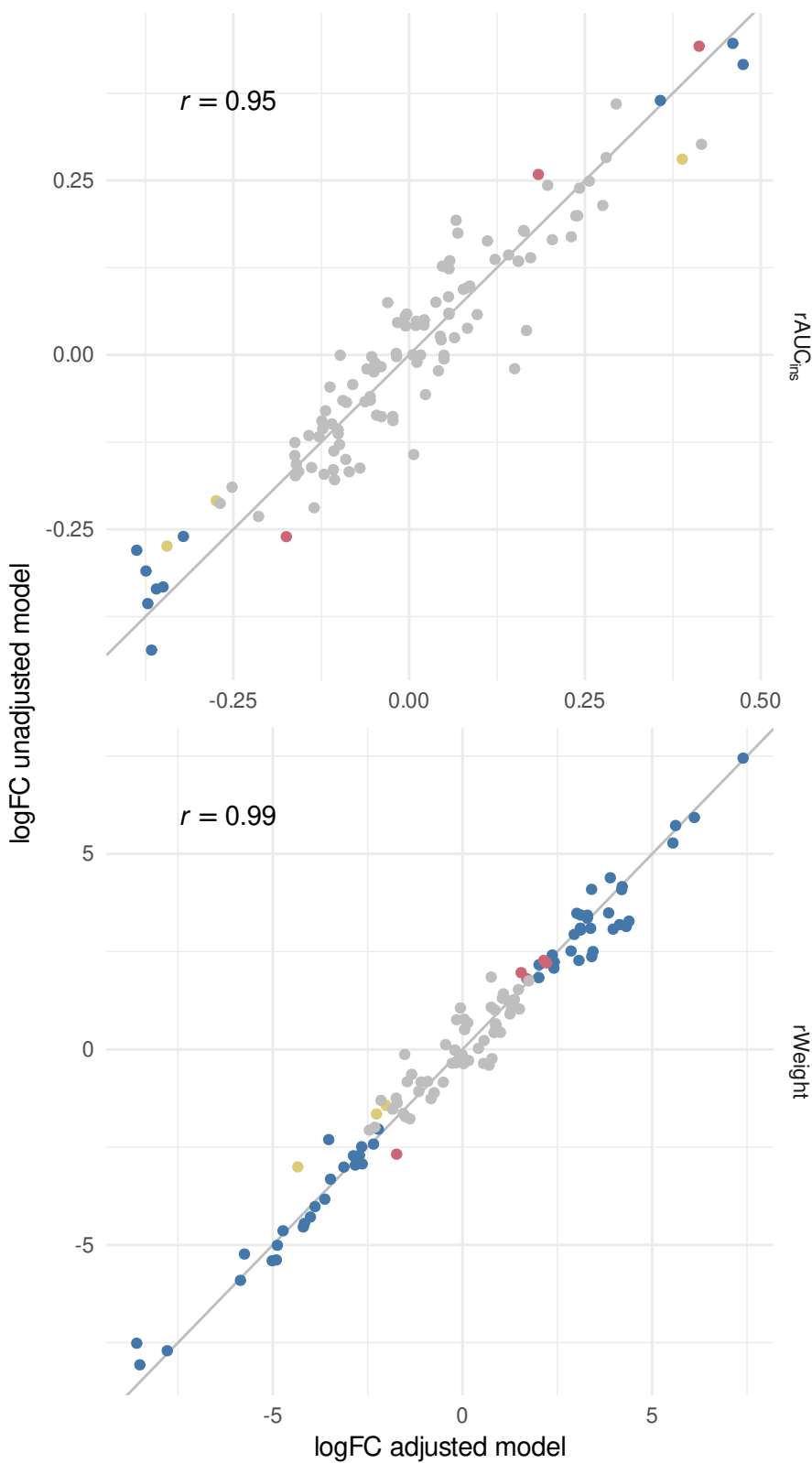

Supplement: Supplemental Information 3 — The adjusted models include both predictors (rAUCins and rWeight), while the unadjusted models include only one them. The almost perfect agreement (high correlation along the identity line, r ≥ 0.95) shows that the models are not affected by collinearity issues between rAUCins and rWeight. [file peerj-09-10764-s003.pdf]

P170168

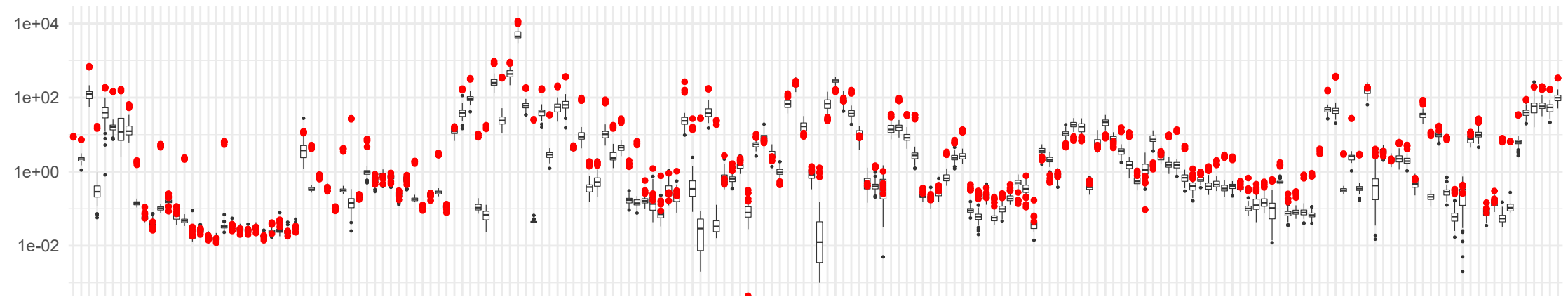

P170169

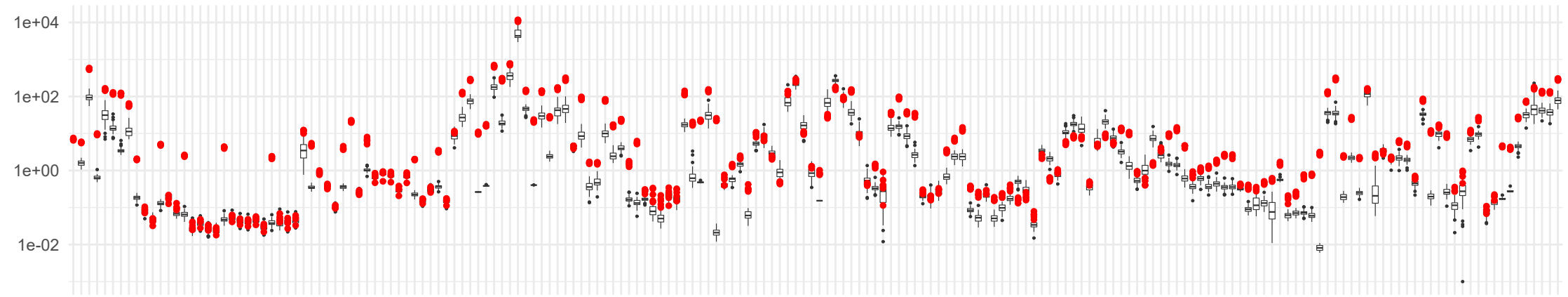

P180534

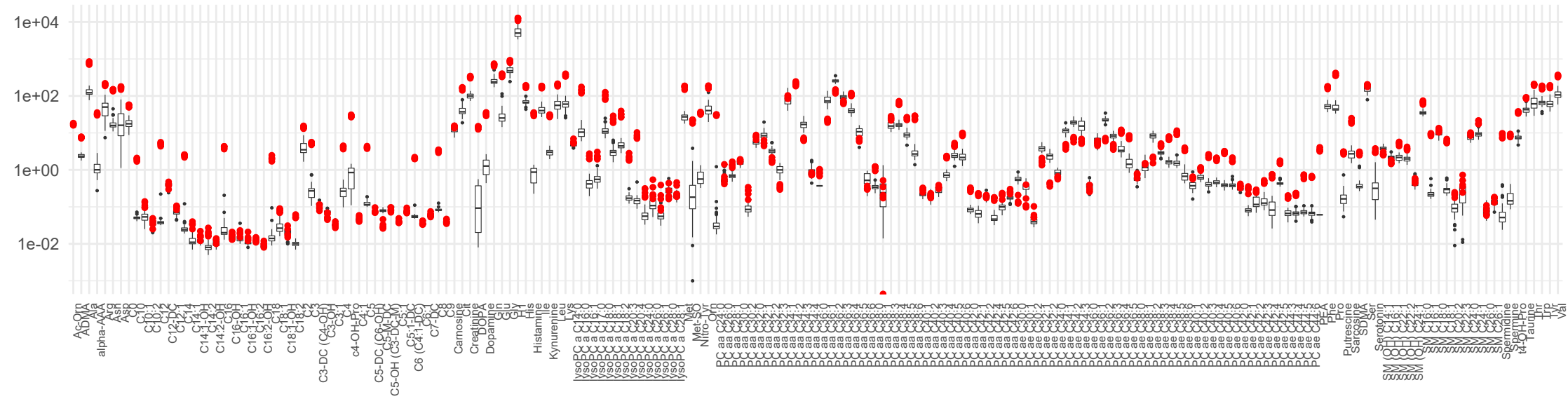

Supplement: Supplemental Information 4 — Boxplots of the raw sample data compared to the medium human QC (lyophilized human plasma with medium concentration levels). The plot is split in three parts (one for each measurement batch/assay plate). The boxplots contain all sample measurements for each metabolite and the human samples (which are technical replicates) are shown on top as red dots. Overall, the patterns are very close among batches and human levels are close to the equine ones a well. [file peerj-09-10764-s004.pdf]
